# Dclk1^+^ small intestinal epithelial tuft cells display the hallmarks of quiescence and self-renewal

**DOI:** 10.18632/oncotarget.5129

**Published:** 2015-09-02

**Authors:** Parthasarathy Chandrakesan, Randal May, Dongfeng Qu, Nathaniel Weygant, Vivian E. Taylor, James D. Li, Naushad Ali, Sripathi M. Sureban, Michael Qante, Timothy C. Wang, Michael S. Bronze, Courtney W. Houchen

**Affiliations:** ^1^ Department of Medicine, University of Oklahoma Health Sciences Center, Oklahoma City, OK, USA; ^2^ Stephenson Oklahoma Cancer Center, University of Oklahoma Health Sciences Center, Oklahoma City, OK, USA; ^3^ Department of Veterans Affairs Medical Center, Oklahoma City, OK, USA; ^4^ Klinikum rechts der Isar, II. Medizinische Klinik, Technische Universität München, Munich, Germany; ^5^ Department of Digestive and Liver Diseases, Columbia University Medical Center, New York, NY, USA; ^6^ COARE Biotechnology, Oklahoma City, OK, USA

**Keywords:** Dclk1, self-renewal, pluripotency, quiescence

## Abstract

To date, no discrete genetic signature has been defined for isolated Dclk1^+^ tuft cells within the small intestine. Furthermore, recent reports on the functional significance of Dclk1^+^ cells in the small intestine have been inconsistent. These cells have been proposed to be fully differentiated cells, reserve stem cells, and tumor stem cells. In order to elucidate the potential function of Dclk1^+^ cells, we FACS-sorted Dclk1^+^ cells from mouse small intestinal epithelium using transgenic mice expressing YFP under the control of the Dclk1 promoter (Dclk1-CreER;Rosa26-YFP). Analysis of sorted YFP^+^ cells demonstrated marked enrichment (~6000 fold) for Dclk1 mRNA compared with YFP^−^ cells. Dclk1^+^ population display ~6 fold enrichment for the putative quiescent stem cell marker Bmi1. We observed significantly greater expression of pluripotency genes, pro-survival genes, and quiescence markers in the Dclk1^+^ population. A significant increase in self-renewal capability (14-fold) was observed in *in vitro* isolated Dclk1^+^ cells. The unique genetic report presented in this manuscript suggests that Dclk1^+^ cells may maintain quiescence, pluripotency, and metabolic activity for survival/longevity. Functionally, these reserve characteristics manifest *in vitro*, with Dclk1^+^ cells exhibiting greater ability to self-renew. These findings indicate that quiescent stem-like functionality is a feature of Dclk1-expressing tuft cells.

## INTRODUCTION

Doublecortin-like kinase 1 protein (Dclk1) is a marker of gastrointestinal tuft cells [1] and has been suggested to mark quiescent stem cells and tumor-initiating stem cells (TSCs) [2, 3]. Rare Dclk1^+^ cells are capable of label retention for at least 10 days following total body irradiation (TBI)-induced injury [4]. Moreover, epithelial Dclk1 plays a critical regulatory role in the epithelial restorative response and is required for satisfactory restitution and survival following severe TBI injury [5]. Recent findings [6] demonstrate that not all Dclk1^+^ cells are equal, but, overall, these cells play a significant role in epithelial homeostasis and may play an even greater role during epithelial injury repair. Although most Dclk1^+^ cells turn over within a few weeks, others remain resident (quiescent) and are among the longest-lived cells within the epithelia [6]. In contrast to earlier reports [1], some Dclk1^+^ tuft cells are independent of the intestinal secretory lineage [7, 8]. While the functional importance of Dclk1 is becoming clearer, it remains complicated by the potential presence of Dclk1^+^ subsets within the epithelial population. Therefore, the functional potential and identity of these morphologically distinct cells remains mysterious. Here we present a discrete gene signature obtained solely from Dclk1-CreER;Rosa26-YFP mice, which will be a valuable animal model for future work seeking to uncover the latent abilities of Dclk1 cells and their subsets.

## RESULTS AND DISCUSSION

We recently reported the phenotype of the Dclk1-CreER;Rosa26-YFP mouse model [6]. We demonstrated that ~5% of Dclk1^+^ cells are long-lived and quiescent. Furthermore, we also confirmed that (i) Dclk1^+^ cells were activated and expanded during intestinal injury, and (ii) were rarely involved in intestinal homeostasis, based on lineage tracing following short-term tamoxifen injection, but actively participated in the response to severe geno/cytotoxic epithelial injury [6]. The present study was designed to address the following fundamental questions: (1) what are the enriched functional candidate genes expressed in isolated Dclk1^+^ cells that are involved in the maintenance of quiescence and/or self-renewal? and (2) do these cells exhibit specific gene expression signs that may explain their longevity and cell survival response to injury?

**Figure 1:**
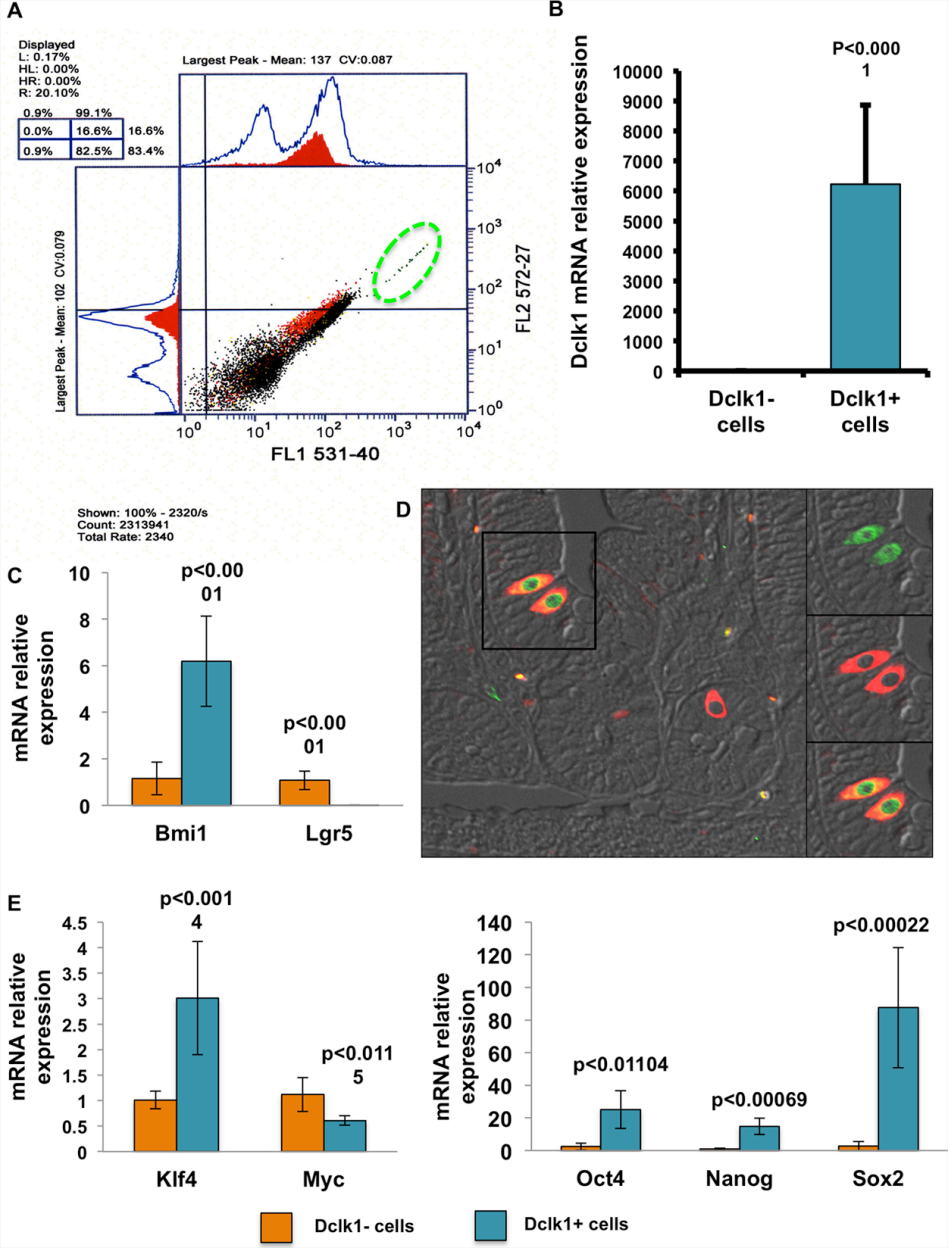
YFP^+^ cells demonstrate enrichment for stem and pluripotency factors **A.** Scatter plot of epithelial cells isolated from the small intestines of Dclk1-CreER;Rosa26-YFP mice and sorted for YFP+/−EpCAM+ and PI− by FACS. **B.** Collected YFP^+^ and YFP^−^ fractions were then subjected to Dclk1 mRNA expression analysis by RT-PCR. YFP+ cells were significantly enriched for Dclk1 mRNA compared with YFP^−^ cells. **C.** mRNA expression analysis via RT-PCR reveals that Dclk1^+^ sorted cells are significantly enriched for stem cell marker Bmi1 but diminished for Lgr5 **D.** Intestinal tissue sections from Dclk1-CreER;Rosa26-YFP mice were stained for anti-Dclk1 (red). Staining of Dclk1 strongly overlapped with YFP^+^ cells in the small intestine. **E.** mRNA expression analysis via RT-PCR reveals that Dclk1^+^ sorted cells are significantly enriched for pluripotency factors Oct 4, Nanog, Sox2, and KLf4, when compared with the Dclk1^−^ cell population. All quantitative data are expressed as means ± *SD* of a minimum of three independent experiments. *P* values of <0.05 were considered statistically significant.

Understanding the molecular mechanisms that regulate activation of signaling pathways and control self-renewal, cell cycle progression, cellular metabolism, and DNA repair is critical for the development of tools to treat gastrointestinal diseases. However, the survival and activation of quiescent stem-like cells is expected to be an important feature for restitution following injury [9–11]. When injected with tamoxifen, the Dclk1-CreER;Rosa26-YFP compound mouse will co-express YFP/Dclk1, which facilitates isolation by FACS within 24 h. YFP^+^ cells account for 0.2–1.4% of all IECs in the Dclk1-CreER;Rosa26-YFP mouse (Figure 1A). This is consistent with the proposed number of quiescent “stem-like cells” among the entire IEC population along the cephalocaudal axis [12, 13]. Analysis of sorted YFP^+^ cells demonstrated marked enrichment (~6000 fold) for Dclk1 mRNA compared with YFP^−^ cells, confirming successful isolation of Dclk1^+^ cells (Figure 1A and 1B). Furthermore, intestinal cross sections from Dclk1-CreER;Rosa26-YFP mice stained positively for YFP; this strongly overlapped with the expression of Dclk1 (Figure 1D). Collected Dclk1^+^ and Dclk1^−^ fractions were analyzed for the quiescent and cycling stem cell markers Bmi1 and Lgr5, respectively. The Dclk1^+^ cell fraction was ~6 fold enriched for Bmi1, whereas the Dclk1^−^ fraction was enriched ~174 fold for Lgr5 (Figure 1C). These findings are consistent with previous lineage tracing studies that demonstrated that Bmi1 and Lgr5 are markers of quiescent and cycling stem cells, respectively [14, 15]. Although Dclk1 and Lgr5 were reported to co-express in a relatively rare population of intestinal epithelial cells [16], the present study indicates that the Dclk1 promoter-derived YFP-expressing cells are instead non-enriched with Lgr5 expression.

**Figure 2:**
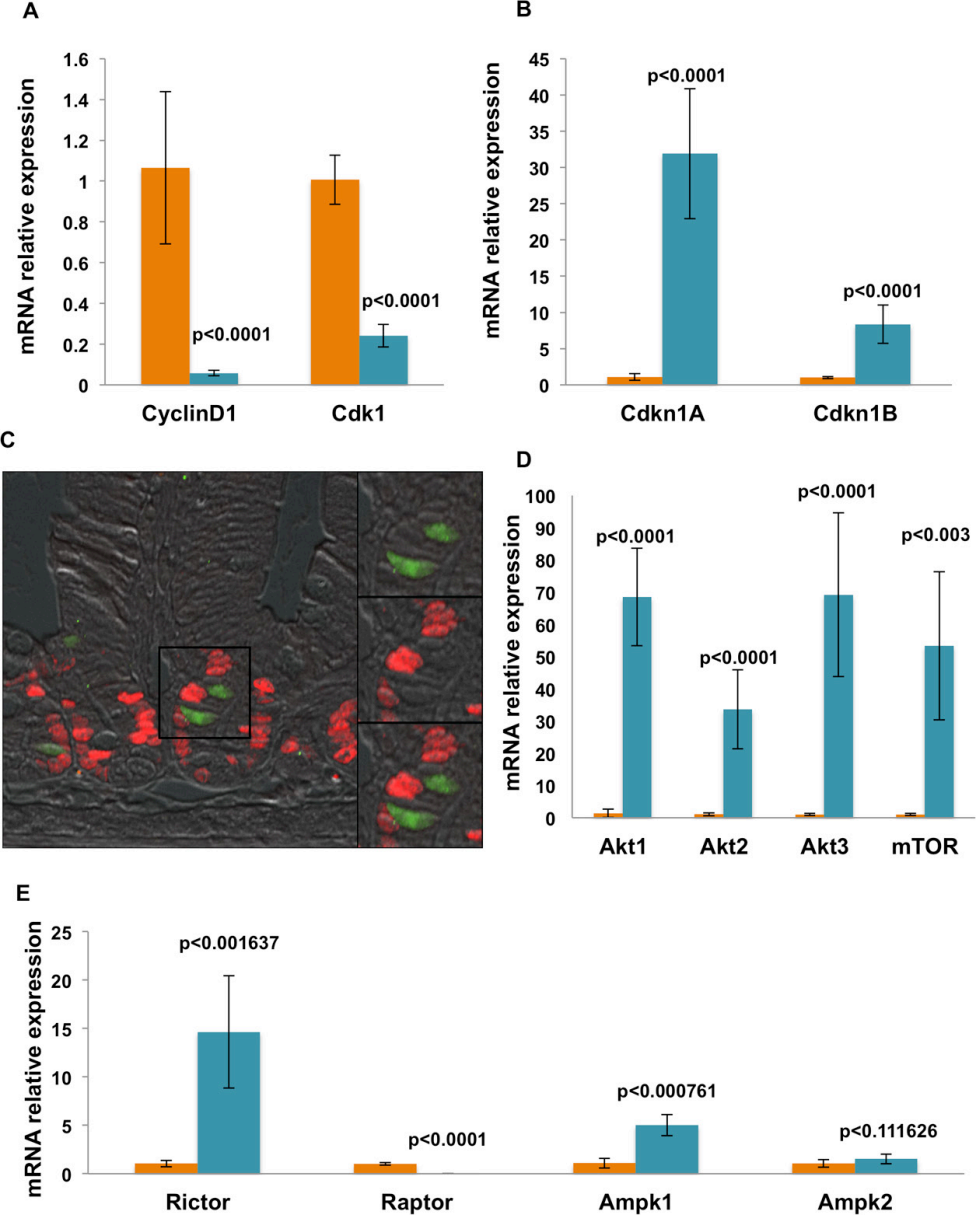
mRNA expression analysis shows that isolated Dclk1^+^ cells are genetically equipped for quiescence, survival, and longevity **A and B.** Sorted YFP^+^ and YFP^−^ cell fractions were analyzed for mRNA expression of cell cycle regulators by RT-PCR. YFP^+^ cells were denuded of cyclinD1 and Cdk1, but were significantly enriched for Cdkn1A (p21) and Cdkn1A (p27). **C.** Intestinal tissue sections from Dclk1-CreER;Rosa26-YFP mice were stained for the proliferation marker Ki-67 (red). No overlap of Ki-67 staining revealed that YFP^+^ cells in the small intestine are non-cycling. Thus, we next examined candidate genes that are involved in cell survival and metabolism. **D.** We found enrichment of Akt1, Akt2, Akt3, and mTOR mRNA expression via RT-PCR in the YFP^+^ fraction. **E.** We also found enrichment of Ampk-related genes (Rictor and Ampk1) in the YFP^+^ fraction. All quantitative data are expressed as means ± *SD* of a minimum of three independent experiments. *P* values of < 0.05 were considered statistically significant.

Factors that regulate self-renewal and pluripotency of human stem cells have been fairly well described [9, 10, 17, 18]. We sought to evaluate the expression levels of key pluripotency factors in the Dclk1^+^ cell population. Pluripotency factors are important in the maintenance of intestinal epithelial self-renewal and can be utilized for epithelial reprogramming of fully differentiated somatic cells [19]. We observed a clear enrichment of mRNA expression of Oct4/Pou5f1, Sox2, Nanog, and Klf4 in Dclk1^+^ cells compared with Dclk1^−^ cells (*p* < 0.01; Figure 1E). Taken together, these data support the hypothesis that Dclk1^+^ tuft cells are enriched for factors that not only favor multipotency, but may also have pluripotent capacity. However, the tightly controlled balance of self-renewal and cell cycling that characterizes normal stem cell function is highly dysregulated during tumorigenesis [20]. Therefore, cells with pluripotency may be the predominant targets in tumor initiation; Dclk1^+^ is one such cell type that has been recently characterized as a tumor stem cell, in colon cancers [2, 6]. To further examine the propensity for proliferation in Dclk1^+^ cells, we next evaluated their cell cycling status by analyzing the expression levels of cell cycle regulatory genes utilizing RT-PCR. In Dclk1^+^ cells, cell cycle initiators, such as cyclinD1 (Ccnd1) and Cdk1 [21], were reduced 18 and 4 fold, respectively (*p* < 0.0001), compared with Dclk1^−^ cells (Figure 2A). Cyclin-dependent kinase (cdk) inhibitors, including the stem cell regulators Cdkn1A (p21) and Cdkn1B (p27), have been widely studied in quiescent and cycling progenitor stem cell models [22–24]. These cell cycle regulators regulate G_0_-/G_1_-S phase transition and cell cycle arrest [22, 25]. In the Dclk1^+^ cells, the expression of Cdkn1A and Cdkn1B was increased 27 and 8 fold, respectively (*p* < 0.0001), compared with Dclk1^−^ cells (Figure 2B). Furthermore, IHC analysis of intestinal cross sections showed that Ki-67, a proliferation marker, did not overlap with Dclk1^+^ (YFP) cells (Figure 2C), consistent with previous reports [26]. These observations support the notion that Dclk1^+^ cells are likely quiescent under basal conditions, but nonetheless express the necessary factors for pluripotency. This enrichment may be required to support the rescue of severely damaged or deleted homeostatic stem cells in response to severe genotoxic injury. This is in accordance with the recent report demonstrating that Dclk1^+^ cells lineage trace after intestinal injury [6].

All cells, and stem cells in particular, must carefully balance their bio-energetic needs to maintain functionality, longevity, damage resistance, and promote survival/growth in response to cellular stress [27, 28]. To determine whether key metabolic pathways were differentially expressed in Dclk1^+^ cells, we first examined the expression of Akt, Ampk, and mTOR. The Akt/Ampk/mTOR signaling pathways are critical for bioenergetic signaling involved in the maintenance of stem cell quiescence, proliferation and differentiation [29]. Akt, Ampk, and mTOR signaling components were enriched within isolated Dclk1^+^ cells when compared with Dclk1^−^ cells (Figure 2D; Figure 2E), suggesting that these cells are metabolically active. We are aware that this may solely be due to the differentiation status or other cell-specific features, and is not necessarily related to stemness. Nevertheless, these findings confirm that Dclk1^+^ cells express the machinery required for metabolic activities. We next looked at Rictor, the activation of which is generally involved in protein biogenesis and regulation of the mRNA transcription machinery to promote cell survival; Raptor is required for cell cycle entry from quiescence [30]. Ampk is an energy sensor kinase involved in energy metabolism and cell cycling, and acts as a negative regulator of Raptor [27, 28, 31]. Isolated Dclk1^+^ cells were enriched for Rictor and Ampk1 at 9 fold and 4.5 fold (*p* < 0.001), with a corresponding low expression of Raptor 54 fold (*p* < 0.0001; Figure 2E), suggesting that Dclk1^+^ cells are metabolically active, but are likely quiescent at baseline.

**Figure 3:**
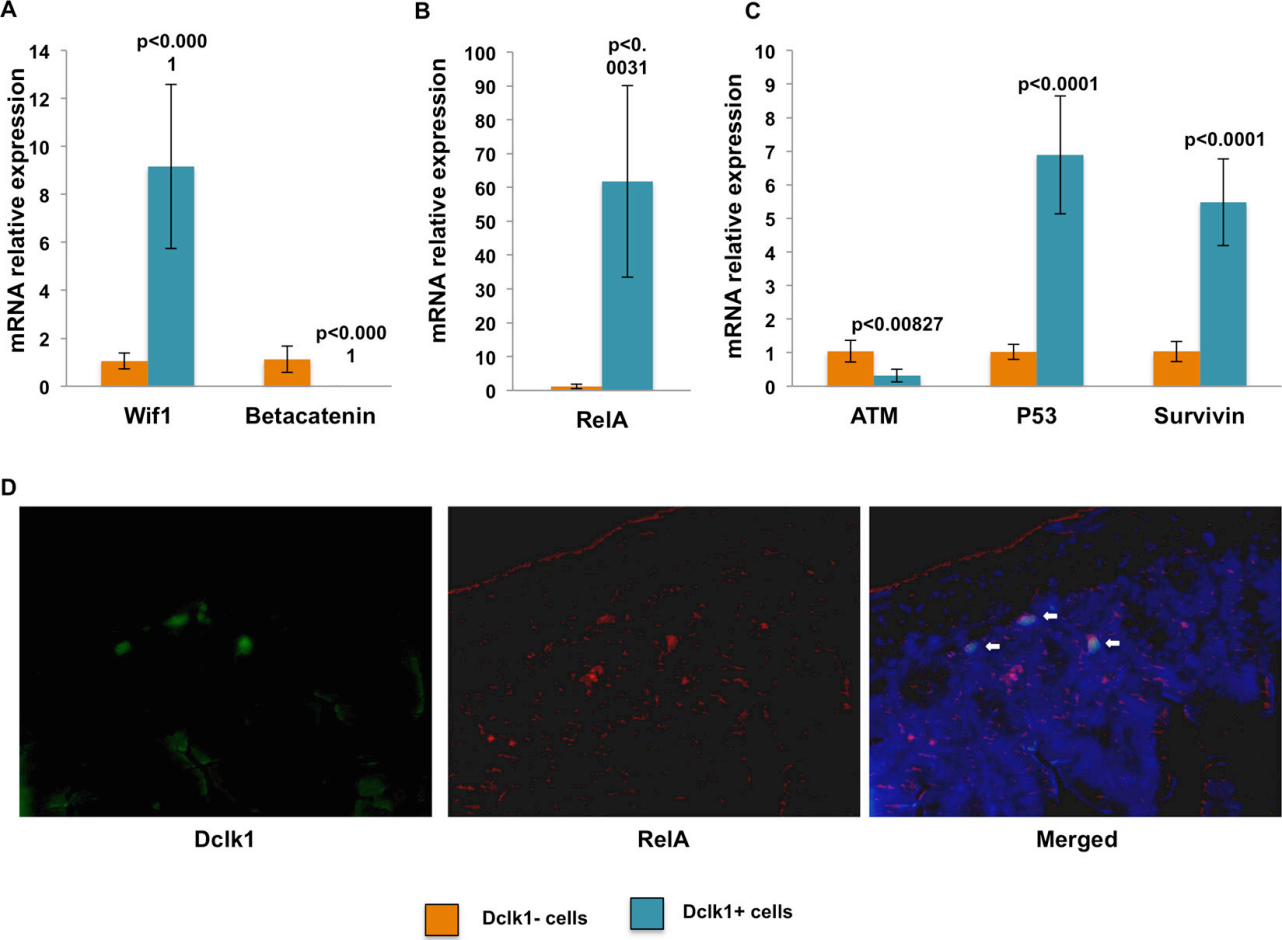
mRNA expression data central to illuminating the potential reserve role of Dclk1^+^ cells **A.** Wnt inhibitory factor 1 (Wif1) is a key factor regulating wnt signaling, including nuclear β-catenin activation. mRNA expression of Wif1 increased, while expression of β-catenin decreased in the isolated YFP^+^ fraction. **B and C.** Ataxia telangiectasia mutated gene (ATM) was decreased in YFP^+^ cells. mRNA analysis in the YFP^+^ fraction showed significant enrichment of p53, survivin, and RelA. **D.** FISH analysis showing the expression of RelA and Dclk1 mRNA in the small intestine of Dclk1-CreER;Rosa26-YFP mice. All quantitative data are expressed as means ± *SD* of a minimum of three independent experiments. *P* values of <0.05 were considered statistically significant.

Next, we evaluated the expression of survival factors and genomic stability machinery, including Atm, Tp53, and Survivin. Atm is preferentially expressed in active cycling cells and regulates self-renewal, but not proliferation or differentiation [32, 33]. In contrast, Atm deletion completely ablated the quiescent stem cell population in mouse bone marrow [34]. Tp53 is a well-known tumor suppressor and anti-proliferative protein; Survivin (Birc5) is a cell cycle inhibitor that supports cell persistence following injury [35, 36]. We observed decreased expression of Atm (~3.2 fold), as would be predicted given its role in proliferation, and enrichment of Survivin (~5.2 fold) and Tp53 (~6.7 fold) in the Dclk1^+^ fraction compared with the Dclk1^−^ fraction (Figure 3C). This finding provides additional evidence that Dclk1^+^ cells are non-cycling, but retain the ability to maintain genomic stability via their enrichment of Tp53 and Survivin. Next, we examined Wnt inhibiting factor1 (Wif1) and RelA expression, key elements for stem cell maintenance and survival [37, 38]. Both were increased in the Dclk1^+^ fraction (~7.2 fold and 61 fold) compared with the Dclk1^−^ fraction (*p* < 0.0001; Figure 3A and 3B). Utilizing FISH analysis we observed a strong expression of RelA mRNA in the Dclk1 expressing cells of the small intestine. Sense probes were used as negative control and no positive signal was observed (Figure 3D). Indeed expression of β-catenin mRNA was significantly reduced (*p* < 0.0001; Figure 3A). However, reduced expression of β-catenin mRNA does not necessarily indicate reduced nuclear β-catenin signaling, as β-catenin signaling is regulated at the protein level. Nevertheless, it is critical for the expansion of stem cells and reduced β-catenin mRNA may lead to less protein available for nuclear translocation and activation of downstream transcriptional activity. Therefore, reduced β-catenin in Dclk1^+^ cells may be consistent with its function as a negative regulator of quiescence [39]. Taken together, these data provide support for the hypothesis that specific gene regulatory targets within the Dclk1^+^ cell population preferentially maintain cells in a prolonged, metabolically stable, quiescent state, but the cells remain prepared for activation by geno/cytotoxic insult. However, in normal cells, these signaling balances are dysregulated in conditions linked to hyperplasia, dysplasia, and neoplasia. Dysregulation of p53 initiates tumor formation by activating the oncogenic pathways and increased pluripotency [40, 41]. Thus, Dclk1^+^ cells may have potential signaling targets by which they transform into tumor stem cells and/or tumor-initiating cells following geno/cytotoxic insult.

**Figure 4:**
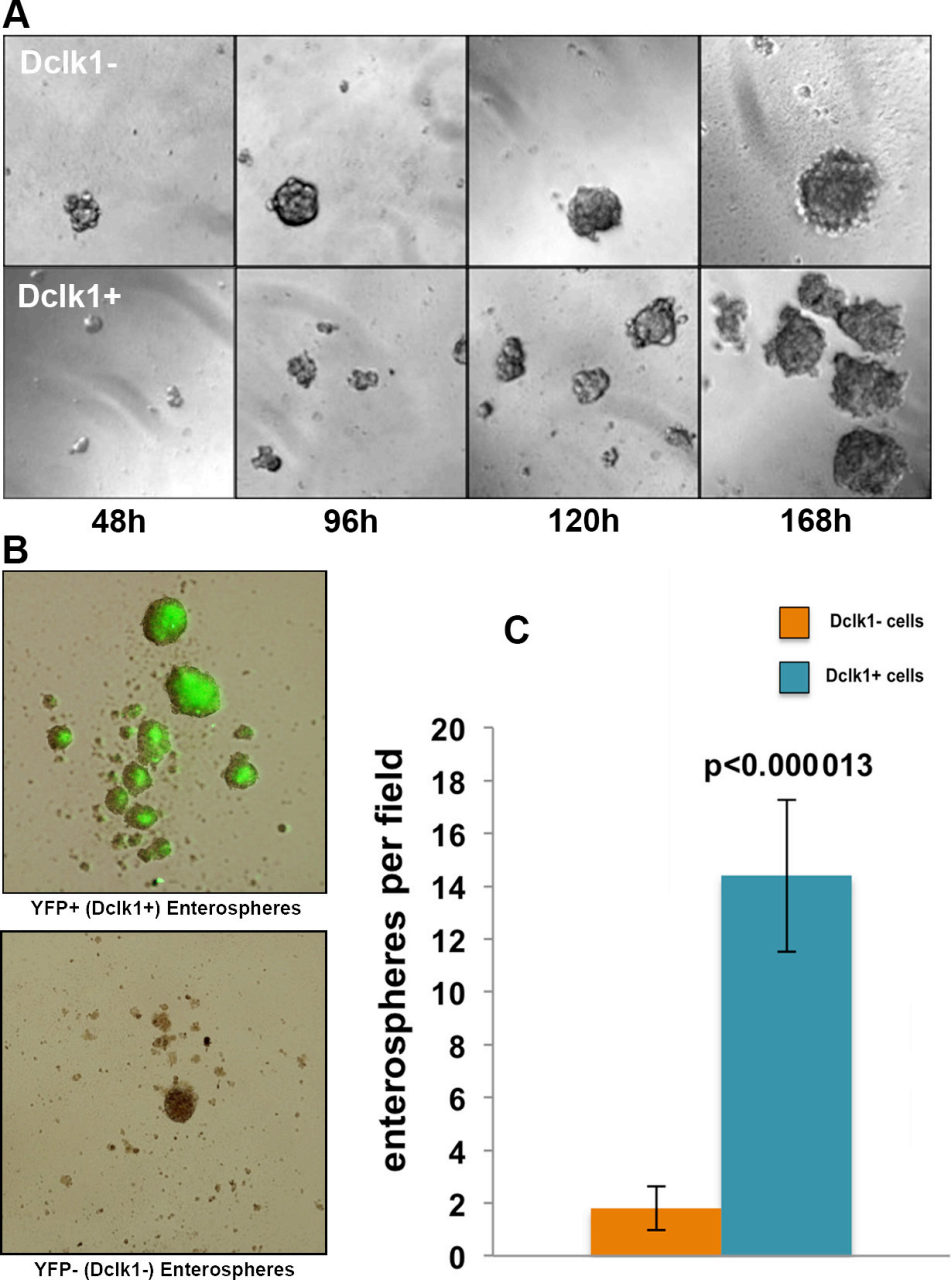
Dclk1 enriched cell population demonstrates significantly greater self-renewal *in vitro* **A.** Dclk1-enriched (YFP^+^ sorted) cells exhibit 14-fold greater enteroid growth potential over 168 hours. **B.** Phase contrast and fluorescent microscopic overlay of YFP^+^ and YFP^−^ enteroids. **C.** Bar graph representing the average number of enterospheres formed per low-power field (original magnifications 100X).

Self-renewal is the hallmark of stem cells/stem-like cells. This ability to self-renew is required to expand and produce daughter cells. Our mRNA transcript data demonstrate that Dclk1^+^ cells are non-cycling and enriched with pluripotency factors. To determine whether Dclk1^+^ cells have latent stem-like features, we next assessed their self-renewal, utilizing an *in vitro* 3-D soft agar clonogenic assay [42]. We chose the soft agar clonogenic assay because this assay only selects cells that retain the ability of secondary and tertiary self-renewal and that can self-renew in primary culture; however, no other normal epithelial cells can survive and/or clonally expand to form spheroids [42]. YFP^+^ and YFP^−^ cells were isolated by FACS from Dclk1-CreER;Rosa26-YFP mice and cultured in soft agar [42, 43]. The Dclk1^+^ cells formed enterospheres by 48 h and budding enteroids within 168 h, representing the self-renewal of stem cell-like properties. Fluorescent imaging confirmed persistent YFP expression within enteroids. Similarly, the Dclk1^−^ cell fraction formed enterospheres somewhat earlier than the Dclk1^+^ fraction. These enterospheres also grew into enteroids within 168 h (Figure 4A and 4B). However, when the overall enteroid growth was quantified, only 1–2% of the Dclk1^−^ fraction displayed the ability to self-renew, as defined by enteroid formation, compared with ~18% in the Dclk1^+^ fraction (Figure 4C). These data suggest that Dclk1^+^ cells retain the ability to self-renew and can act as stem-like cells, independent of a crypt niche environment. Interestingly, only 18% of the Dclk1^+^ cells exhibited this self-renewal under these culture conditions. Recently, it was reported that a distinct population of Dclk1^+^ cells gives rise to different populations of intestinal cells under homeostasis and this restitution capacity increased after injury [6]. The present study suggests that the population of self-renewable Dclk1^+^ cells is involved in lineage trace under homeostasis and after injury, and may be the cells of origin for tumors. The molecular features of non-self-renewing Dclk1^+^ cells will require further examination, which is beyond the scope of the current manuscript. These observations, however, suggest a potential subset of Dclk1 cells that lack stem-like features.

## SUMMARY AND CONCLUSION

In summary, the cellular signatures that define the intestinal stem cell phenotype are just beginning to be explored. Candidate genes under investigation include those that regulate the maintenance of pluripotency, proliferation status, genomic stability, cellular survival, and a broad array of genetic regulatory signals, which are likely to differ from more differentiated cell types [44, 45]. As part of the injury repair response, de-differentiation of a committed cell towards a pluripotent stem phenotype may occur along with the activation of new molecular programs. A distinct cellular identity for quiescent and cycling stem cells, before and after injury, continues to be an area of intense exploration, with tumor-initiating cells contributing to the complexity of this field of study.

**Figure 5:**
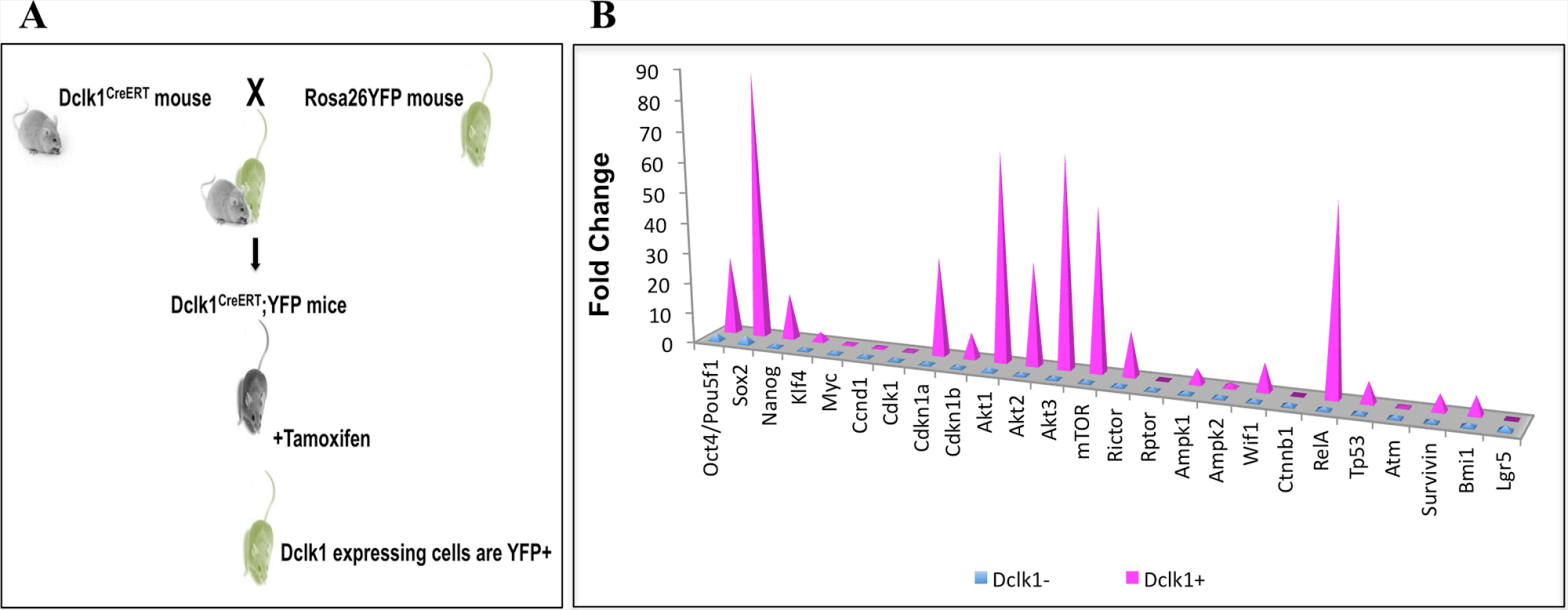
Outline of Dclk1-CreER;Rosa26-YFP mice and clustered mRNA transcripts **A.** Schematic diagram showing the generation of Dclk1-CreER;Rosa26-YFP transgenic mice. **B.** Clustered column chart displays values in 3-D pyramid, values compared across Dclk1-positive and negative cell categories.

Furthermore, the functional characteristics of tuft cells have only recently been investigated. Recent studies have examined Dclk1^+^ chemosensing tuft cells that initiate and trace tumors in APC^min/+^ mice [2]. Dclk1-CreER;Rosa26-YFP mice represent a valuable new tool with which to study the discrete molecular features and functional aspects of Dclk1^+^ cells within the intestinal epithelium (Figure 5A) [6]. The unique genetic profile presented in this manuscript suggests that Dclk1^+^ cells may maintain quiescence, pluripotency, and metabolic activity for survival/longevity through the expression of Cdkn1A, Cdkn1B, Oct4, Sox2, Nanog, Klf4, Wif1, RelA, Akt and AMPK, and vital regulation of Raptor, Rictor, p53, and Survivin (Figure 5B). Functionally, these reserve characteristics manifest *in vitro*, with Dclk1^+^ cells exhibiting greater self-renewal ability. Taken as a whole, these results provide additional support for the hypothesis that while the majority of Dclk1^+^ cells are committed tuft cells, they also maintain the molecular capability for stemness in reserve, and could play a role in the maintenance of the stem cell niche in an injury repair environment.

## MATERIALS AND METHODS

### Animals

All animal experiments were performed with the approval and authorization of the Institutional Review Board and Institutional Animal Care and Use Committee at the University of Oklahoma Health Sciences Center (Oklahoma City, Oklahoma). Dclk1-CreER mice were crossed with Rosa26-YFP mice to generate Dclk1-CreER;Rosa26-YFP compound heterozygotes. Adult mice were intraperitoneally injected with tamoxifen (2 mg per 40 g of body weight; Sigma) to label the Dclk1 cells. Forty-eight hours after tamoxifen injection, small intestines were dissected and used for the isolation of intestinal epithelial cells (IEC).

### Intestinal epithelial cell (IEC) isolation

Small intestines were attached to a paddle, immersed in Ca^2+^-free standard Krebs-buffered saline (in mmol/liter: 107 NaCl, 4.5 KCl, 0.2 NaH_2_PO_4_, 1.8 Na_2_HPO_4_, 10 glucose, and 10 EDTA) at 37°C for 15–20 min, and gassed with 5% CO_2_, 95% O_2_. Individual crypt units were then separated by intermittent (30 sec) vibration into ice-cold phosphate-buffered saline and collected by centrifugation. The pellets were washed with phosphate-buffered saline, suspended in RPMI glutamax medium/0.5 U/ml dispase at 37°C, and shaken gently for 5–10 min. The cells were pelleted and suspended again in RPMI glutamax medium for FACS analysis [42, 46–48].

### FACS

The isolated IEC pellet was suspended in 10 ml of PBS and filtered through a 40 μm cell strainer (BD Bioscience) to remove debris and clumps; the strainer was further cleaned with an additional 10 ml of PBS. The filtered suspension was centrifuged 5 min at 1000 rpm. The isolated cells were suspended in HBSS 10% FCS. To isolate the pure population of YFP+ live epithelial cells, isolated cells were incubated with anti-EpCAM antibodies for 30 min and propidium iodide (PI) before sorting. The cells were washed and sorted using an Influx-V cell sorter (Cytopeia) [43]. YFP^+^EpCAM^+^PI^−^ cells were collected and subjected to molecular analysis.

### Antibodies

The following antibodies were utilized: EpCAM, Ki-67, Dclk1 (all from Abcam, Cambridge, MA), Alexa Fluor 488 Donkey Anti-Rabbit IgG and Alexa Fluor® 568 Donkey Anti-Goat IgG (from Life Technologies, IL, USA).

#### Immunohistochemistry

Heat-induced epitope retrieval was performed on 4-μm formalin-fixed, paraffin-embedded sections utilizing a pressurized Decloaking Chamber (Biocare Medical LLC, Concord, CA) in citrate buffer (pH 6.0) at 99°C for 18 minutes. Fluorescence: Slides were incubated in a normal serum and BSA blocking step at room temperature for 20 minutes. After incubation with primary antibody overnight at 4°C, slides were labeled with Alexa Fluor dye-conjugated secondary antibody and mounted with ProLong Gold (Invitrogen).

#### Fluorescence *in situ* hybridization

FISH was performed as described previously [49] on paraffin-imbedded tissues from Dclk1-CreER;Rosa26-YFP mice according to the protocol of the manufacturer (Stellaris RNA FISH, Biosearch Technologies, CA, USA). RNA probes for Dck1 and RelA were synthesized and the probes were labeled using Alexa Fluor 488 or Alexa Fluor 594 fluorophores labeling kits (Life Technologies, IL, USA). Probes at the concentration of 100 ng/ml were hybridized for a total time of 16 h using the hybridization buffer. Counterstaining was performed prior to mounting with DAPI for 20 min at room temperature, and then sections were imaged.

#### Image acquisition

Slides were examined on the Nikon Eclipse Ti motorized microscope paired with the DS-Fi2 color and CoolSnap ES2 monochrome digital cameras utilizing DIC enhanced PlanApo objectives operated by the NIS-Elements Microscope Imaging Software platform (Nikon Instruments, Melville, NY).

### RNA isolation and real-time (RT)-PCR analysis

The total RNA isolated from YFP^+^ epithelial cells was subjected to reverse transcription. The complementary DNA (cDNA) was subsequently used to perform real-time PCR with SYBR™ chemistry (Molecular Probes, Eugene, OR) using gene-specific primers for specific transcripts. The crossing threshold value assessed by real-time PCR was noted for the transcripts and normalized to β-actin.

### Clonogenic assay

Isolated Dclk1^+^ and Dclk1^−^ cells were plated at a density of 100 cells per well in 48-well plates in RPMI medium containing 0.3% soft agar with 1% fetal calf serum. The cell suspensions were plated in a 48-well plate above a layer of solidified 1% soft agar in plain RPMI medium. The plates were incubated at 37°C under 5% CO_2_. The cells were followed for enterospheres and enteroid formation as described previously [42, 43].

### Statistical analysis

Means with standard deviation (*SD*) are shown in most graphs. All experiments were performed independently a minimum of three times. Some were conducted a maximum of five times. The data were analyzed using the Student's *t*-test to compare mean values between groups. *P* values <0.05 were considered statistically significant.

## Abbreviations

Ampk: Adenosine Monophosphate-Activated Protein Kinase
Apc: Adenomatous polyposis coli
Atm: Ataxia telangiectasia mutated
Bmi1: B-lymphoma Moloney murine leukemia virus insertion region-1
Cdkn: Cyclin-dependent kinase (cdk) inhibitor
cDNA: Complementary DNA
Dclk1: Doublecortin-like Kinase1
FACS: Fluorescence-activated cell sorting
IEC: Intestinal Epithelial cell
IHC: Immunohistochemistry
Klf4: Kruppel-Like factor 4
Lgr5: Leucine-rich repeat-containing G-protein coupled receptor 5
mTOR: Mammalian Target of Rapamycin
Oct4: Octamer-binding transcription factor 4
Sox2: Sry-related HMG box
TBI: Total body irradiation
TSC: Tumor stem cell
Wif1: Wnt inhibiting factor1
YFP: Yellow fluorescence protein
